# White blood cells and type 2 diabetes: A Mendelian randomization study

**DOI:** 10.1371/journal.pone.0296701

**Published:** 2024-03-01

**Authors:** Yaru Bi, Yuan Gao, Yao Xie, Meng Zhou, Zhiyuan Liu, Suyan Tian, Chenglin Sun

**Affiliations:** 1 Department of Endocrinology and Metabolism, First Hospital of Jilin University, Changchun, China; 2 Department of Clinical Nutrition, First Hospital of Jilin University, Changchun, China; 3 Department of Obstetrics and Gynecology, Shengli Oilfield Central Hospital, Dongying, China; 4 Department of Clinical Medicine, Yanbian University, Yanji, China; 5 Division of Clinical Research, First Hospital of Jilin University, Changchun, China; Muhimbili University of Health and Allied Sciences School of Medicine, UNITED REPUBLIC OF TANZANIA

## Abstract

**Background:**

Observational studies have demonstrated an association between white blood cells (WBC) subtypes and type 2 diabetes (T2D) risk. However, it is unknown whether this relationship is causal. We used Mendelian randomization (MR) to investigate the causal effect of WBC subtypes on T2D and glycemic traits.

**Methods:**

The summary data for neutrophil, lymphocyte, monocyte, eosinophil, and basophil counts were extracted from a recent genome-wide association study (n = 173,480). The DIAGRAM and MAGIC consortia offered summary data pertaining to T2D and glycemic characteristics, including fasting glucose (FG) (n = 133,010), glycosylated hemoglobin (HbA1c) (n = 46,368), and homeostatic model assessment-estimated insulin resistance (HOMA-IR) (n = 37,037). A series of MR analyses (univariable MR, multivariable MR, and reverse MR) were used to investigate the causal association of different WBC subtypes with T2D and glycemic traits.

**Results:**

Using the inverse-variance weighted method, we found one standard deviation increases in genetically determined neutrophil [odd ratio (OR): 1.086, 95% confidence interval (CI): 0.877–1.345], lymphocyte [0.878 (0.766–1.006)], monocyte [1.010 (0.906–1.127)], eosinophil [0.995 (0.867–1.142)], and basophil [0.960 (0.763–1.207)] were not causally associated with T2D risk. These findings were consistent with the results of three pleiotropy robust methods (MR-Egger, weighted median, and mode-based estimator) and multivariable MR analyses. Reverse MR analysis provided no evidence for the reverse causation of T2D on WBC subtypes. The null causal effects of WBC subtypes on FG, HbA1c, and HOMA-IR were also identified.

**Conclusions:**

WBCs play no causal role in the development of insulin resistance and T2D. The observed association between these factors may be explained by residual confounding.

## Introduction

Type 2 diabetes (T2D) is a complex and multifactorial metabolic disease characterized by insulin resistance and insufficient insulin secretion. Growing evidence indicates that chronic inflammation is also involved in T2D pathogenesis [1]. Low-grade chronic inflammation is a process of the activation of immune system and the increased circulating cytokines and chemokines, wherein the adipose tissue appears to be a major source of pro-inflammatory factors [2]. Chronic inflammation might promote the occurrence of T2D by increasing insulin resistance, affecting insulin signaling, and promoting beta-cell dysfunction [3–5].

Multiple inflammation markers could reflect the low-grade chronic inflammation process, such as white blood cell (WBC) and its subtype, tumor necrosis factor-α, and interleukin-6 [6, 7]. Of them, WBC is routinely measured in clinical practice. Circulating WBCs comprise granulocytes (neutrophils, eosinophils, and basophils), lymphocytes, and monocytes, which may be biologically linked with T2D through inflammation and insulin resistance. Briefly, neutrophils, the most abundant WBC subtype, accounting for 50–70% of circulating WBCs, are one of the first immune cells to migrate into the adipose tissue, indicating their role in adipose tissue inflammation and insulin resistance [6, 8, 9]. Lymphocytes, an important adaptive immune cell type, could infiltrate into the expanding adipose depots, especially in obese individuals, and produce cytokines and chemokines, promoting chronic inflammation, insulin resistance, and diabetes development [9, 10].

Several observational studies have reported that elevation in the WBC subtypes, such as neutrophils and lymphocytes, is associated with T2D risk [11–14]. For example, one meta-analysis, including 20 observational studies, indicated that neutrophil and lymphocyte counts in the top tertile increased the risk of T2D compared to the bottom tertile [relative ratio: 1.58 (95% CI: 1.09–2.29) vs. 1.06 (1.02–1.56), respectively] [12]. However, the causal association between the two attributes could not be ascertained as the findings could have been affected by confounding factors. Furthermore, reverse causality could not be excluded in observational studies. For example, hyperglycemia might enhance myelopoiesis in the bone marrow, leading to elevated levels of WBCs, such as neutrophils and monocytes [15, 16].

Mendelian randomization (MR) is an approach used for causal inference. It involves using genetic variants (single nucleotide polymorphisms, SNPs) reliably associated with exposure as instrumental variables (IVs) to estimate the causal effect of exposure (such as neutrophil count) on a trait or disease (such as T2D). Given the random inheritance and nonmodifiable nature of genetic variants, MR analysis can help reduce the bias caused by confounders or reverse causation. To our knowledge, only three studies have explored the causal association between WBC and glucose metabolism. Borné et al used a R262W polymorphism (rs3184504) as an instrumental variable (IV) to determine the causal effect of total WBC on fasting glucose (FG), glycosylated hemoglobin A1c (HbA1c), and diabetes using one-sample MR analysis [14]. Astle et al. explored the causal effect of correlated-13 blood cell traits (including platelet indices, red cell indices, and white cell indices) on multiple complex diseases, one of which was T2D [17]. Another recent MR study by Li et al. investigated the causal association of WBC, its five subtypes, and lymphocyte subtypes with T2D using the univariable MR analysis [18]. However, Borné et al. used only one SNP as IV, which may have insufficient statistical power. Furthermore, much is still unknown about the causal association between WBC and glucose metabolism. For example, considering the apparent correlations among WBC subtypes, the independent causal effect of WBC subtypes on T2D using multivariable MR analysis has not been assessed. Moreover, whether 5 WBC subtypes are causally associated with glycemic traits and whether the predisposition to T2D affects the circulating levels of WBC subtypes are both unknown.

In the current study, we used MR to comprehensively investigate the relationship between genetically determined neutrophil, lymphocyte, monocyte, eosinophil, and basophil counts and T2D liability and glycemic traits, including individual univariable MR analyses, multivariable MR, sand reverse MR. Such studies could help researchers better understand the role of WBC subtypes in glucose metabolism and T2D pathogenesis.

## Material and methods

### Experimental data

First, we investigated the effects of genetic variants strongly associated with circulating neutrophil, lymphocyte, monocyte, eosinophil, and basophil as IVs to investigate their effects on T2D and glycemic traits. We also performed reverse MR analyses to assess the effect of T2D predisposition on neutrophil, lymphocyte, monocyte, eosinophil, and basophil counts to rule out the possibility of reverse causation. The study was a second-analysis based on publicly available genome-wide association study (GWAS) data. No additional ethical approval was needed.

A GWAS with data from 173,480 European ancestors from the UK biobank and INTERVAL studies was used to extract summary statistics for the association between SNPs and circulating WBC subtypes [17]. To reduce the influence of noisy blood counts on the genetic associations, the participants with blood cancer or major blood disorders, such as leukemia, lymphoma, and multiple myeloma, were excluded from the UK Biobank. The inclusion of blood disorders is likely to be substantially lower as the participants in the INTERVAL study were active whole-blood donors. Further details can be found in the reference [17].

The DIAGRAM consortium provided data on the association between SNPs and T2D, which included data from 12 different cohort studies with 12,171 cases and 56,862 controls of European ancestry [19]. The MAGIC consortium’s summary data for FG (n = 133,010) [20], HbA1c (n = 46,368) [21], and homeostatic model assessment-estimated insulin resistance (HOMA-IR) (n = 37,037) [22] were obtained. The individuals are of European ancestry and do not have diabetes. It is noted that HbA1c (in percentage) was measured using the National Glycohemoglobin Standardization Program (NGSP)-certified method in the HbA1c GWAS study from 31 cohorts to decrease the errors from the HbA1c measurement [21].

Independent SNPs (linkage disequilibrium R^2^ < 0·01, window distance = 5,000kb) associated with circulating neutrophils, lymphocytes, monocytes, eosinophils, and basophils were defined as genetic instruments for WBC subtypes at genome-wide levels of significance (P < 5×10^−8^). Furthermore, to improve the accuracy of the causal estimation, we excluded exposure-related SNPs that were not present in the outcome GWAS. In addition, we harmonized the effect alleles to identify the risk alleles associated with the exposure and the outcome on the same strand. In the reverse MR analysis, we used multiple independent SNPs (R^2^ < 0·01) strongly associated with T2D at genome-wide levels of significance (P < 5×10^−8^) from the DIAGRAM consortium, with the harmonization process done in the above analysis. F-statistics was calculated to evaluate the power of SNPs using the formula beta^2^/se^2^. SNPs with less statistical power (F-statistics < 10) were removed in the final analyses.

### Statistical analyses

The conventional inverse-variance weighted (IVW) method was used for causal estimation in the univariable MR analysis. In addition, as supplements to IVW, the other three robust methods MR-Egger, weighted-median, and weighted-mode were used. Cochrane’s Q statistics were used to detect SNP heterogeneity. When SNPs showed heterogeneity, IVW with multiplicative random effect was used. The pleiotropy was detected using the MR-Egger intercept and the MR-pleiotropy residual sum and outlier (MR-PRESSO) method (the distribution was set 1,000 times). MR-PRESSO assisted in detecting outliers. If outliers were found, the specified SNPs would be removed in order to reassess the causal estimate. A leave-one-out analysis was used to see if a single SNP has a significant impact on the relationship between exposure and outcome. We also used multivariable MR analyses with three different models to assess the independent causal effect of WBC subtypes on T2D. Model 1 included neutrophil, lymphocyte, and monocyte counts, while model 2 included eosinophil and basophil counts. The five circulating WBC subtypes were considered in model 3. The IVW was used as the primary method, and MR-Egger was used as complementary method. The R package “TwoSampleMR” was used to performed the univariable MR analysis. MR-PRESSO was conducted using the R package “MRPRESSO”. Multivariable MR was performed using the R package “MendelianRandomization”. All statistical analyses were performed in R software 4.1.0 (https://www.r-project.org/). R software and GraphPad Prism are used to create the artwork.

## Results

### Univariable MR analyses

We aimed to investigate the causal effect of the levels of WBC subtypes on T2D incidence. In the univariable MR analysis, 63, 83, 91, 65, and 32 SNPs were used as IVs for neutrophil, lymphocyte, monocyte, eosinophil, and basophil counts, respectively. The F statistics ranged from 29.81 to 1280.97, indicating that the causal estimations were not biased by the weak IVs. Detailed information on the IVs used in the MR analyses was displayed in S1 Table.

**Fig 1:**
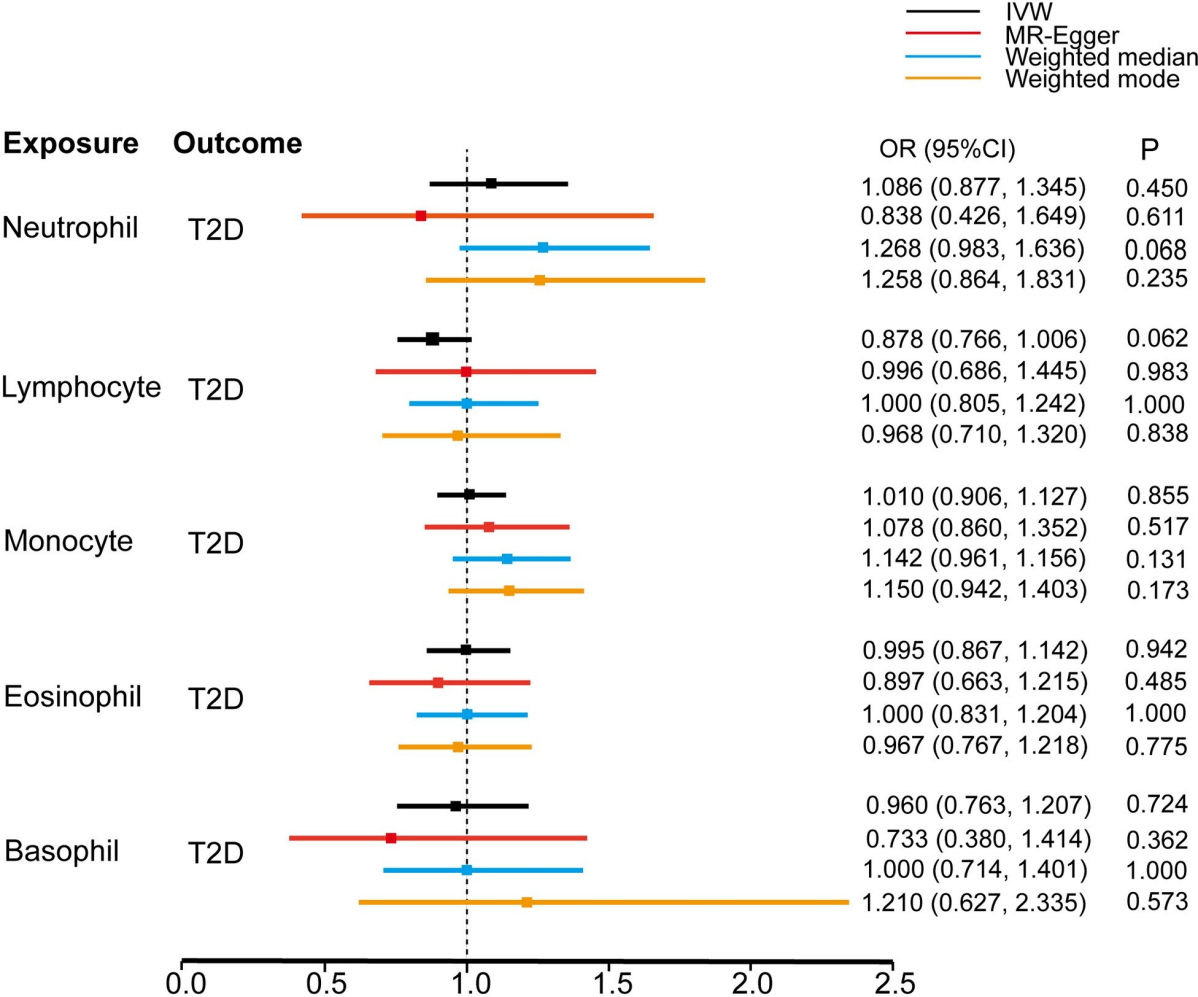
Estimate of the univariable MR analysis for the white blood cells and risk of T2D.

**Fig 2:**
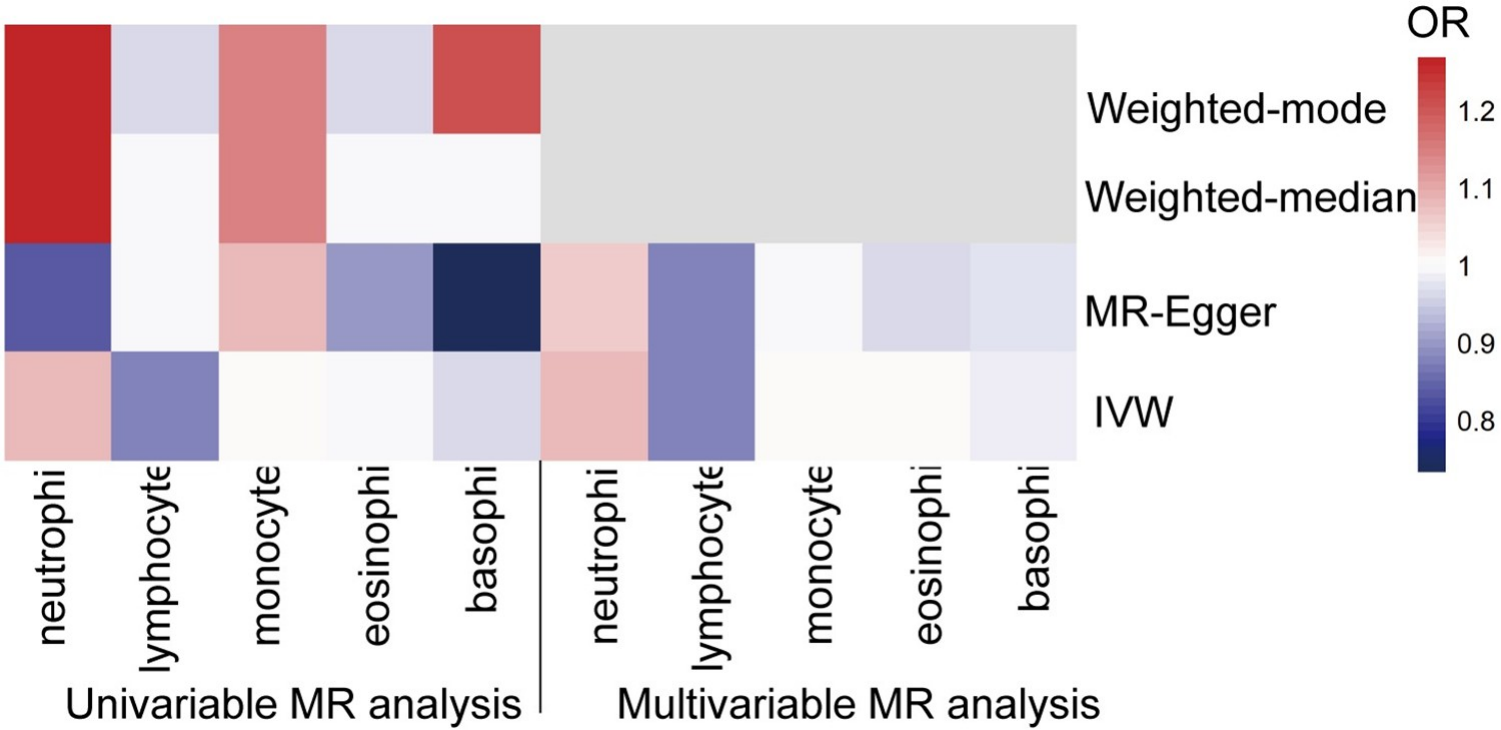
MR analyses testing the effects of five WBC subtypes on T2D. The result of univariable MR obtained using four MR approaches (IVW, MR-Egger, Weighted-median, and weighted mode) and multivariable MR obtained using two MR approaches (IVW and MR-Egger) of Model 3. Causal estimates were presented as a heatmap. All results indicated no statistically significance.

The odds ratios (ORs) of T2D development for a 1-standard deviation (SD) increase in genetically determined neutrophil, lymphocyte, monocyte, eosinophil, and basophil counts were 1.086 (95% CI: 0.877–1.345, P = 0.450), 0.878 (0.766–1.006, P = 0.062), 1.010 (0.906–1.127, P = 0.855), 0.995 (0.867–1.142, P = 0.942) and 0.960 (0.763–1.207, P = 0.724), respectively, according to the IVW estimates. Three pleiotropy robust MR approaches, including MR-Egger, weighted-median, and weighted-mode, also identified the null causal association (Figs 1 and 2).

The results of the heterogeneity and pleiotropy tests are listed in Table 1. We found potential heterogeneities in the analyses of neutrophil and eosinophil counts, and outliers for lymphocyte count. We adapted random-IVW or removed the outliers to reassess the causal estimate. The forest plots, scatter plots, and funnel plots are shown in S1 Fig. The results of the leave-one-out analysis indicated that no SNP significantly altered the IVW association (S1 Fig).

**Table 1:** Results of heterogeneity and pleiotropy tests of the causal effect of WBC on T2D.

Exposure	Outcome	NSNP	F-statistics	heterogeneity		pleiotropy	
			Mean (min-max)	methods	Cochran’s Q statistics (P)	MR-Egger intercept (P)	MR-PRESSO outliers
Neutrophil	T2D	63	57.40 (30.77–301.76)	MR-Egger	98.76 (0.002)	0.008 (0.432)	No SNP
				IVW	99.77(0.002)		
Lymphocyte	T2D	83	66.64 (29.87–606.35)	MR-Egger	79.23 (0.535)	-0.005 (0.477)	2SNPs (rs13431440, rs3818717)
				IVW	79.74 (0.550)		
Monocyte	T2D	91	104.67 (30.75–1280.97)	MR-Egger	92.06 (0.391)	-0.003 (0.522)	No SNP
				IVW	92.49 (0.408)		
Eosinophil	T2D	65	110.76 (29.90–821.43)	MR-Egger	85.93 (0.030)	0.005 (0.454)	No SNP
				IVW	86.70 (0.031)		
Basophil	T2D	32	62.96 (29.81–209.21)	MR-Egger	28.85 (0.525)	0.009 (0.399)	No SNP
				IVW	29.58 (0.539)		

Abbreviation: T2D, type 2 diabetes; SNP, single-nucleotide polymorphism; IVW, inverse variance weighted; MR-PRESSO, MR pleiotropy residual sum and outlier

**Fig 3:**
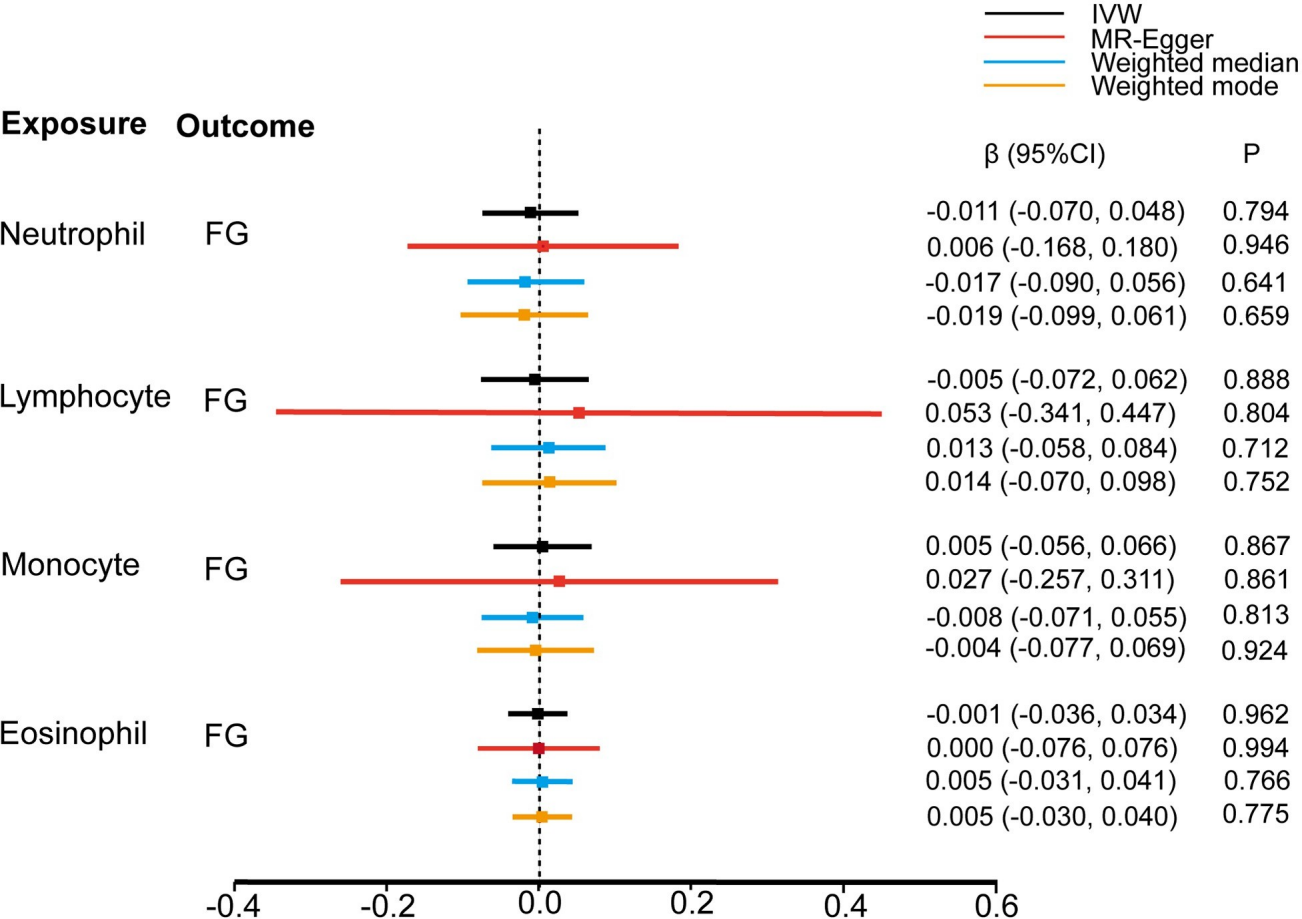
Estimate of the MR analysis for the white blood cells and FG.

**Fig 4:**
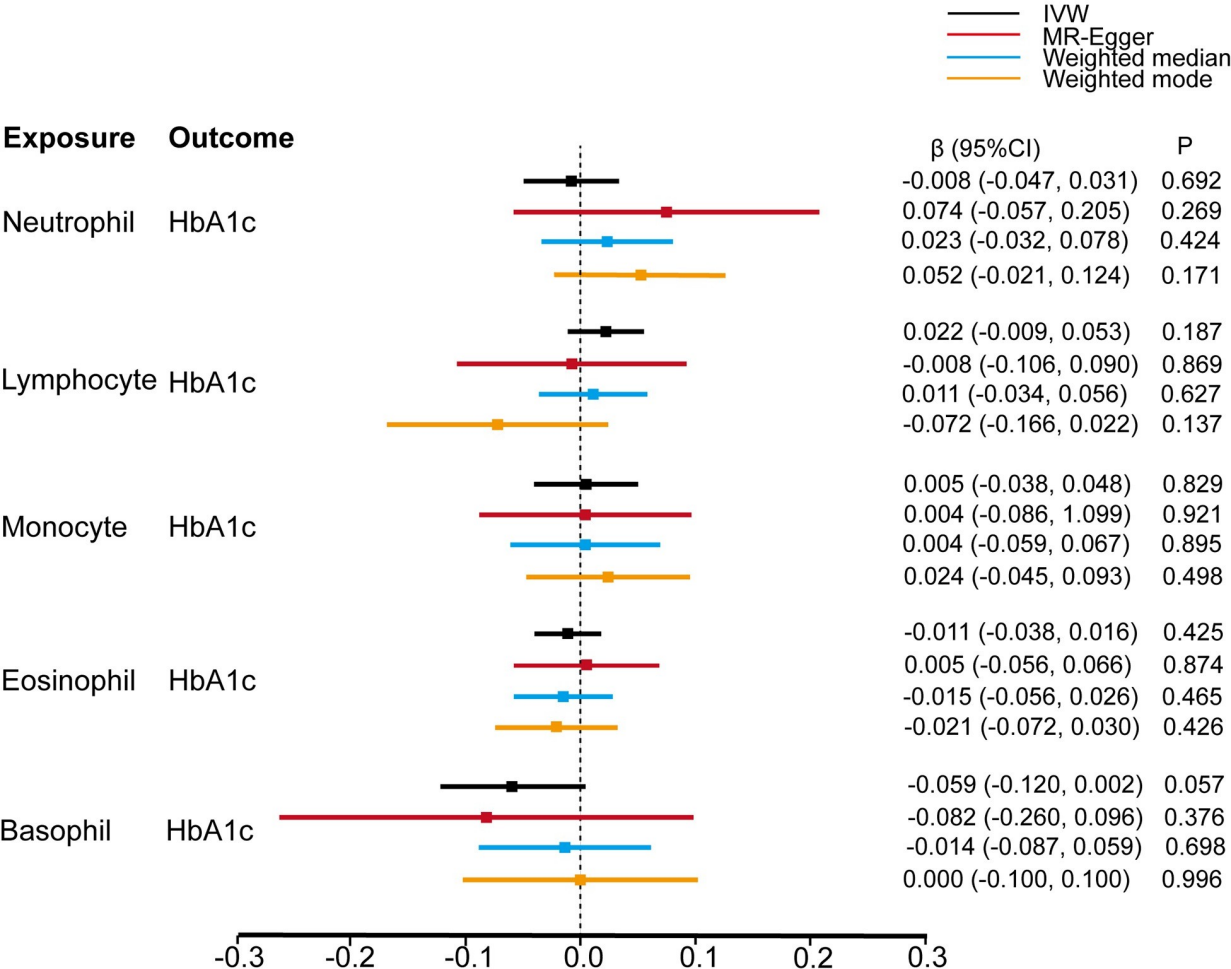
Estimate of the MR analysis for the white blood cells and HbA1c.

**Fig 5:**
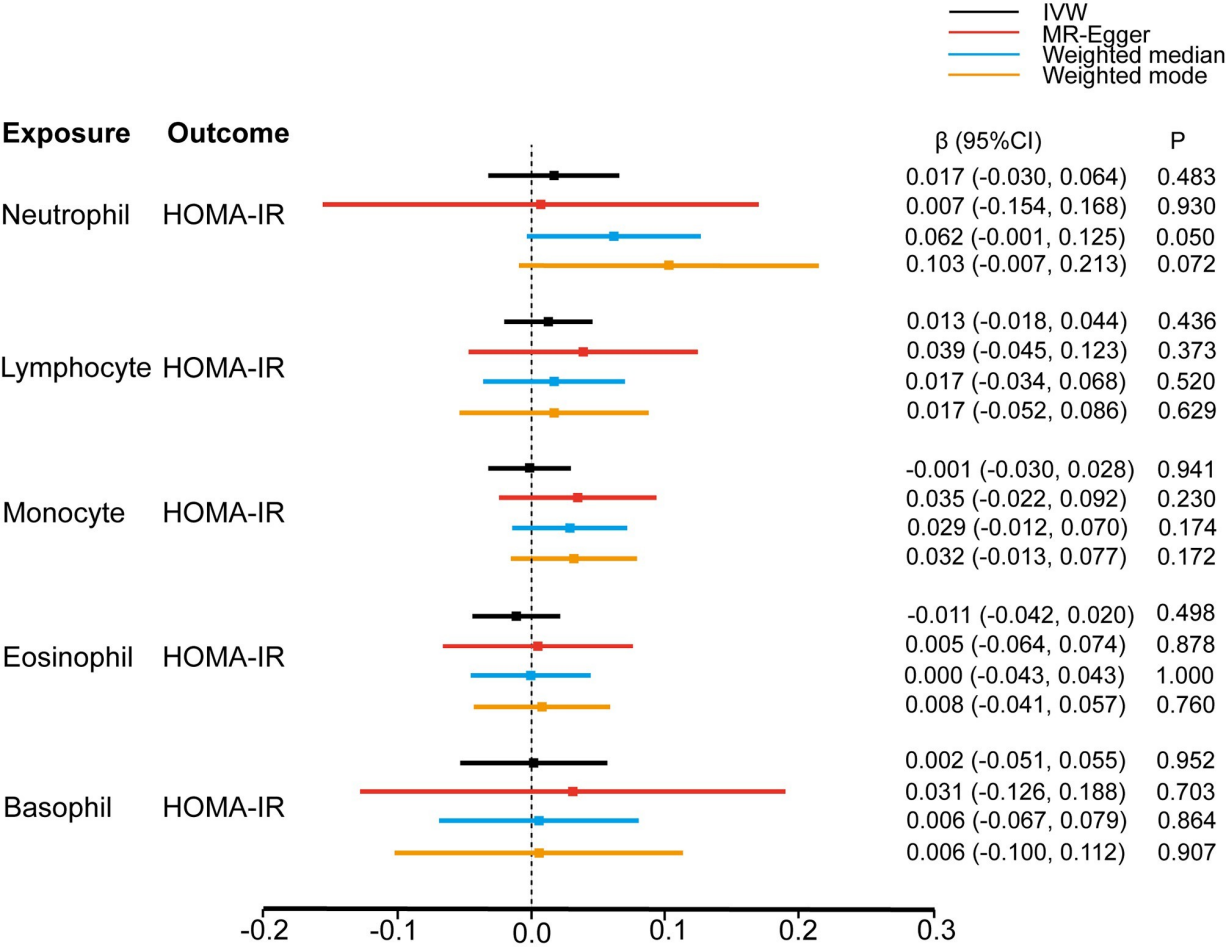
Estimate of the MR analysis for the white blood cells and HOMA-IR.

**Fig 6:**
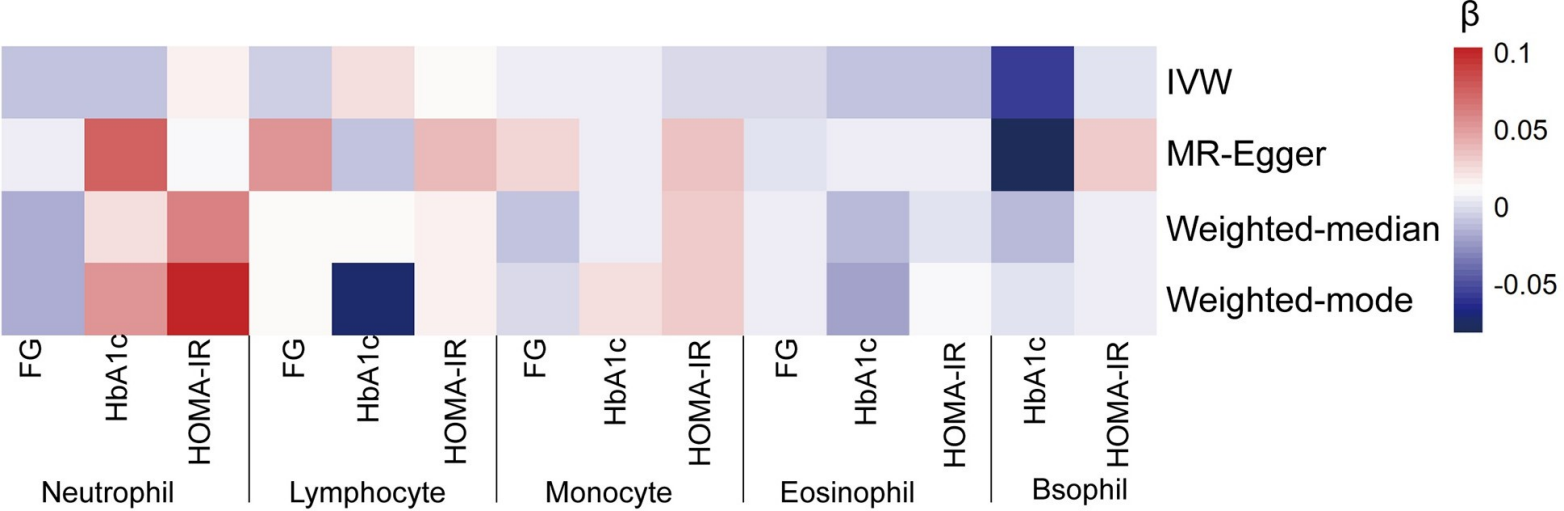
MR analyses testing the effect of five WBC subtypes on glycemic traits. Results obtained using 4 MR approaches (IVW, MR-Egger, Weighted-median and weighted-mode) were presented as a heatmap representing causal estimate. All results indicated no statistically significance.

Further, we assessed the causal effect of the counts of different WBC subtypes on the glycemic traits of individuals without diabetes. Based on the conventional IVW methods, we found that genetically predicted neutrophil, lymphocyte, monocyte, eosinophil, and basophil counts (1-SD increase) were not associated with FG (Fig 3), HbA1c (Fig 4), and HOMA-IR (Fig 5). These findings were consistent with the results obtained with the other three MR approaches. The causal estimates were presented as heatmap (Fig 6). We found heterogeneities in the analyses of HbA1c by neutrophil, lymphocyte, and basophil count and in the analyses of HOMA-IR by neutrophil, monocyte, and eosinophil counts. No significant horizontal pleiotropy was detected in any of the MR analyses (S2 Table). The results of the leave-one-out analyses suggested that none of the SNPs dramatically altered the results. Notably, only two SNPs existed in the causal estimate of basophil count on FG. The causal estimate between basophil counts and FG was not shown in Fig 3.

### Multivariable MR analyses in model 1, model 2, and model 3

**Fig 7:**
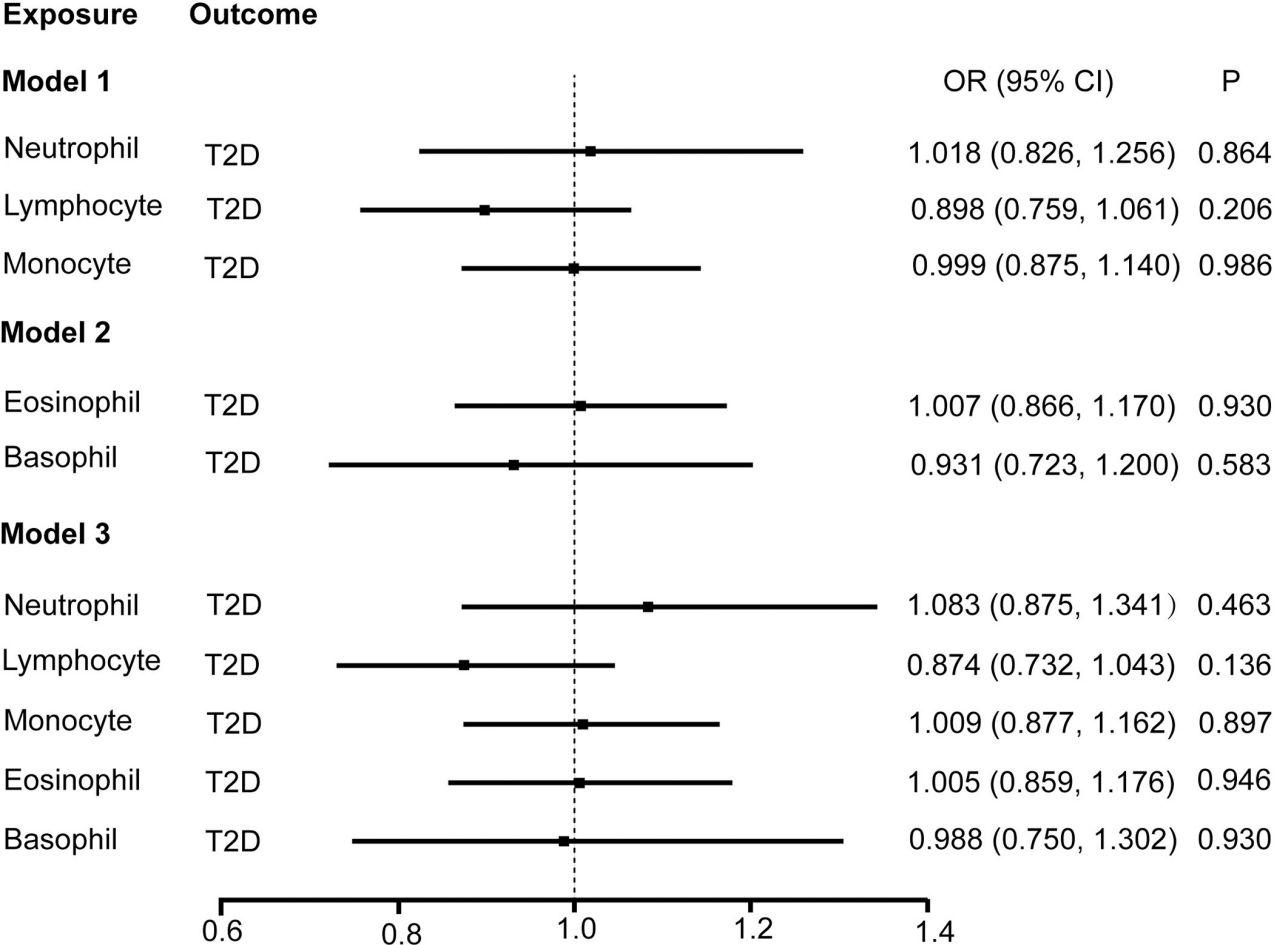
Estimate of the multivariable MR analysis for white blood cells and risk of T2D. Model 1, association of neutrophil, lymphocyte, and monocyte with T2D risk. Model 2, association of eosinophil and basophil with T2D risk. Model 3, association of neutrophil, lymphocyte, monocyte, eosinophil and basophil with T2D risk.

Multivariable MR analyses were performed to assess the independent effect of WBC subtypes on T2D incidence. A total of 182, 87, and 222 SNPs were involved in the model 1, 2 and 3, respectively. In model 1, neutrophil, lymphocyte, and monocyte counts were jointly used as exposures and T2D as the outcome. The results indicated that neutrophil [1.018 (0.826–1.256), P = 0.864], lymphocyte [0.899 (0.759–1.061), P = 0.206], and monocytes [0.999 (0.875–1.140), P = 0.986] were not causally associated with T2D risk after controlling for the other two predictors. In model 2, the null causal associations were identified of both eosinophil and basophil with T2D after controlling for basophil or eosinophil, respectively. In model 3, which incorporated five WBC subtypes, we found no causal association of neutrophil [1.083 (0.875–1.341), P = 0.463], lymphocyte [0.874 (0.732–1.043), P = 0.136], monocyte [1.009 (0.877–1.162), P = 0.897], eosinophil [1.005 (0.859–1.176), P = 0.946], and basophil [0.988 (0.750–1.302), P = 0.930] with T2D development after controlling for the other four exposures (Fig 7). Adoption of the MR-Egger method also suggested no genetic evidence of the independent causal effect of any of the WBC subtypes on T2D incidence in the three models. The causal estimates of the multivariable MR analyses using the IVW and MR-Egger methods are presented as a heatmap in Fig 2.

### Reverse MR analyses

**Fig 8:**
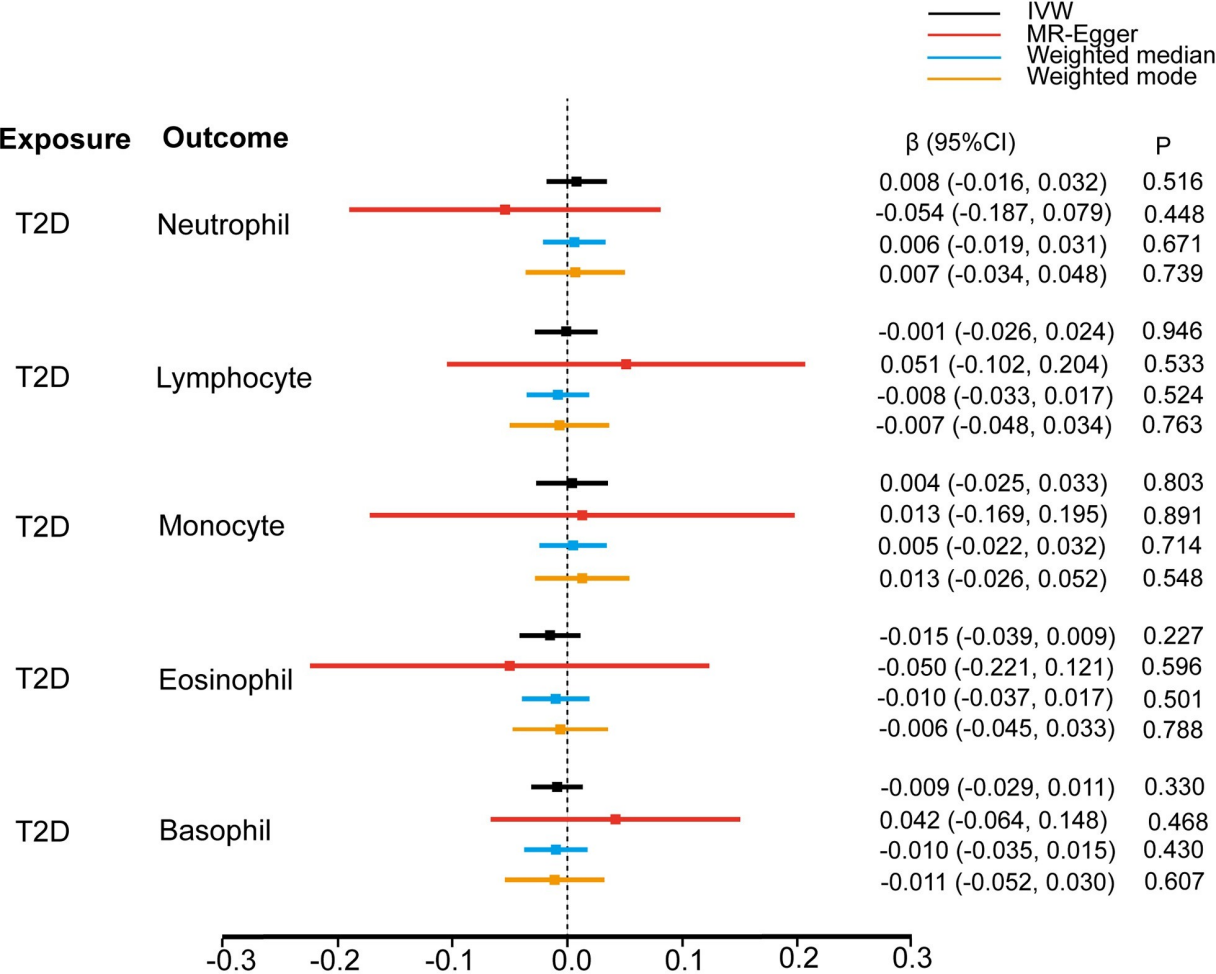
Reverse MR analysis of the causal effect of T2D on white blood cells.

To further understand the source of observational associations between WBC subtypes and T2D, we also explored the relationship of T2D with WBC subtypes in the reverse direction using reverse MR analyses. Overall, the results of reverse MR analyses did not support the reverse causation of T2D predisposition on the levels of any of the WBC subtypes. In the main IVW analysis, neutrophil and monocyte counts in the T2D group were, respectively 0.008 SD (-0.016, 0.032) and 0.004 SD (-0.025, 0.033) higher than those in the non-diabetes group. Further, the lymphocyte, eosinophil, and basophil counts were, respectively 0.001 SD (-0.026, 0.024), 0.015 SD (-0.039, 0.009), and 0.009 SD (-0.029, 0.011) lower than those in the non-diabetes group (Fig 8). We observed heterogeneities in the lymphocyte and monocyte counts. The random-IVW results suggested null causal associations. No horizontal pleiotropy was detected in the reverse MR (Table 2). Detailed information on the IVs used in the reverse MR analyses were displayed in S3 Table.

**Table 2:** Results of the heterogeneity and pleiotropy tests of the causal effect of T2D on WBC.

Exposure	Outcome	NSNP	F-statistics	heterogeneity		pleiotropy	
			Mean (min-max)	methods	Cochran’s Q statistics (P)	MR-Egger intercept (P)	MR-PRESSO outliers
T2D	Neutrophil	9	48.95 (30.80–100.00)	MR-Egger	12.14 (0.096)	0.009 (0.380)	1 SNP
							(rs7651090)
				IVW	13.66 (0.091)		
T2D	Lymphocyte	9	48.48 (30.84, 100.00)	MR-Egger	14.42 (0.044)	-0.0073 (0.519)	1 SNP
							(rs864745)
				IVW	15.37 (0.052)		
T2D	Monocyte	8	48.82 (30.80, 100.00)	MR-Egger	17.30 (0.008)	-0.001(0.921)	3 SNPs
							(rs4506565
							rs7651090
							rs864745)
				IVW	17.33 (0.015)		
T2D	Eosinophil	6	39.73 (30.80–48.78)	MR-Egger	1.03 (0.905)	0.005 (0.701)	4 SNPs
							(rs1109077
							rs5015480
							rs864745
							rs9368222)
				IVW	1.21 (0.944)		
T2D	Basophil	8	48.82 (30.80, 100.00)	MR-Egger	3.584 (0.733)	-0.007 (0.372)	2 SNPs
							(rs7651090
							rs864745)
				IVW	4.516 (0.719)		No SNP

Abbreviations: T2D, type 2 diabetes; SNP, single-nucleotide polymorphism; IVW: inverse variance weighted; MR-PRESSO:MR- pleiotropy residual sum and outlier.

## Discussion

In the current study, we investigated the causal association of WBC subtypes with T2D risk and glycemic traits. After analysis of the published GWAS summary data using both the univariable and multivariable MR, we found that genetically predicted neutrophil, lymphocyte, monocyte, eosinophil, and basophil counts were not causally associated with a higher risk of T2D. Reverse MR analyses also provided no evidence for the causal effect of T2D predisposition on five WBC subtypes. Furthermore, we observed no causal association between WBC subtypes and glycemic traits, including FG, HbA1c, and HOMA-IR, in individuals without diabetes.

Several observational studies have suggested that elevated neutrophil and lymphocyte counts are associated with a higher T2D risk [11–13]. However, our MR analyses did not support the causal effect of neutrophil and lymphocyte counts on T2D incidence. Reverse MR analyses further revealed null reverse causation. Therefore, the association of neutrophil and lymphocyte counts with T2D observed in observational studies might be attribute to residual confounders, such as pro-inflammatory cytokines produced by adipose tissue, which are not adjusted in observational studies.

Monocyte was not found to be associated with T2D risk in observational studies [12, 13, 23]. Consistent with these results, we also found no causal link between monocyte count and T2D risk. Most monocytes are attracted to the specific peripheral tissue such as adipose tissue by various stimuli and differentiate into macrophages, which are, in turn, considered the main cells responsible for the association between inflammation, insulin resistance, and T2D [24]. Circulating monocyte counts may not reflect the levels of macrophages in tissues. Whether the macrophage counts in the specific tissue are causally associated with T2D deserves further exploration.

Apart from assessing the causal association between WBC subtypes and T2D, we also investigated the causal effect of genetically determined WBC subtypes on glucose traits. In individuals without diabetes, we found no evidence of the causal link between the combined genetic IVs for WBC subtypes and FG, HbA1c, or HOMA-IR levels. Overall, our results indicated that WBC subtypes play no causal role in the development of abnormal glucose metabolism. We postulated that WBC subtypes might act as simple indicators of chronic inflammatory response observed in insulin resistance and T2D. We further assessed the possible residual confounders in observational studies that might be responsible for the null causal association between WBC subtypes and T2D, as well as insulin resistance in MR. The pro-inflammatory cytokines, such as TNF-α and IL-6, produced by adipose tissue and obesity might act as the initiating factors in inflammatory response (the “driver” role), which might then activate various signal transduction cascades in the cells of insulin responsive tissues, leading to inhibition of insulin signaling, insulin resistance, and T2D [25–29]. WBC subtypes are simply indicators (the “passenger” role) that reflect systematic inflammation.

The study had several advantages. First, the design of MR helps decrease potential confounders and eliminate reverse causality. Second, to the best of our knowledge, the study is the first to assess the reverse causality of T2D on WBC subtypes and the causal effect of WBC subtypes on FG, HbA1c, and HOMA-IR. Third, the implementation of multivariable MR analysis in the current study offered a significant advantage. For one thing, by employing multivariable MR, we devised three distinct models to thoroughly assess the interaction between each WBC subtype and T2D, and for another, given the correlation among the five WBC subtypes, multivariable MR analysis allowed for improved control of the SNP-related measured confounding factors to a certain degree; thus, offering better control for horizontal pleiotropy compared to univariable MR.

The study had several limitations. First, the individuals from both the exposure and the outcome groups were of European ancestry. Therefore, our results still need to be validated in other populations of non-European ancestry. Second, we could not conduct a specific sensitivity or stratified analysis (e.g., based on age category) as we used publicly available summary data. Third, though our MR analysis included data from a considerable number of participants, the number of participants was still too small to detect a subtle difference. Last, although WBCs infiltrating adipose tissue represent a small fraction of circulating WBCs, they contribute to adipose tissue inflammation, insulin resistance, and subsequently, the development of T2D. However, the counts of WBC subtypes in circulation may not accurately reflect their levels within adipose tissue.

## Conclusion

In conclusion, in the current study, which used MR analyses to comprehensively investigate the causal association between circulating WBC subtypes and T2D, we found null causal associations between these two factors. Our results suggested that observational findings of the possible association between WBC subtypes and T2D risk might be attributed to unmeasured confounding. Further research involving diverse ethnic populations is required to explore the potential role and underlying mechanisms of WBC subtypes in relation to T2D.

## Supporting information

S1 Fig Forest plot, scatter plot, funnel plot, and leave-one-out analysis of univariable MR analysis for the causal effect of WBC subtypes on T2D. (PDF)

S1 Table Detailed information on the IVs used in the MR analyses for the causal effect of WBC subtypes on T2D. (XLS)

S2 Table Heterogeneity and pleiotropy test of the causal effect of white blood cells on FPG, HbA1c, and HOMA-IR. (DOC)

S3 Table Detailed information on the IVs used in the MR analyses for the causal effect of T2D on WBC subtypes. (XLS)

## References

[1] Rohm T V, Meier D T, Olefsky J M, and Donath M Y. Inflammation in obesity, diabetes, and related disorders. Immunity, 2022. 55(1): p. 31–55. doi: 10.1016/j.immuni.2021.12.013 35021057 PMC8773457

[2] Kawai T, Autieri M V, and Scalia R. Adipose tissue inflammation and metabolic dysfunction in obesity. Am J Physiol Cell Physiol, 2021. 320(3): p. C375–c391. doi: 10.1152/ajpcell.00379.2020 33356944 PMC8294624

[3] Suren Garg S, Kushwaha K, Dubey R, and Gupta J. Association between obesity, inflammation and insulin resistance: Insights into signaling pathways and therapeutic interventions. Diabetes Res Clin Pract, 2023. 200: p. 110691. doi: 10.1016/j.diabres.2023.110691 37150407

[4] Szukiewicz D. Molecular Mechanisms for the Vicious Cycle between Insulin Resistance and the Inflammatory Response in Obesity. Int J Mol Sci, 2023. 24(12). doi: 10.3390/ijms24129818 37372966 PMC10298329

[5] Cerf M E. Beta Cell Physiological Dynamics and Dysfunctional Transitions in Response to Islet Inflammation in Obesity and Diabetes. Metabolites, 2020. 10(11). doi: 10.3390/metabo10110452 33182622 PMC7697558

[6] Herrero-Cervera A, Soehnlein O, and Kenne E. Neutrophils in chronic inflammatory diseases. Cell Mol Immunol, 2022. 19(2): p. 177–191. doi: 10.1038/s41423-021-00832-3 35039631 PMC8803838

[7] Germolec D R, Shipkowski K A, Frawley R P, and Evans E. Markers of Inflammation. Methods Mol Biol, 2018. 1803: p. 57–79. doi: 10.1007/978-1-4939-8549-4_5 29882133

[8] Michailidou Z, Gomez-Salazar M, and Alexaki V I. Innate Immune Cells in the Adipose Tissue in Health and Metabolic Disease. J Innate Immun, 2022. 14(1): p. 4–30. doi: 10.1159/000515117 33849008 PMC8787575

[9] Zatterale F, Longo M, Naderi J, Raciti G A, Desiderio A, Miele C, et al. Chronic Adipose Tissue Inflammation Linking Obesity to Insulin Resistance and Type 2 Diabetes. Front Physiol, 2019. 10: p. 1607. doi: 10.3389/fphys.2019.01607 32063863 PMC7000657

[10] McLaughlin T, Ackerman S E, Shen L, and Engleman E. Role of innate and adaptive immunity in obesity-associated metabolic disease. J Clin Invest, 2017. 127(1): p. 5–13. doi: 10.1172/JCI88876 28045397 PMC5199693

[11] Zhang H, Yang Z, Zhang W, Niu Y, Li X, Qin L, et al. White blood cell subtypes and risk of type 2 diabetes. J Diabetes Complications, 2017. 31(1): p. 31–37. doi: 10.1016/j.jdiacomp.2016.10.029 27863973

[12] Gkrania-Klotsas E, Ye Z, Cooper A J, Sharp S J, Luben R, Biggs M L, et al. Differential white blood cell count and type 2 diabetes: systematic review and meta-analysis of cross-sectional and prospective studies. PLoS One, 2010. 5(10): p. e13405. doi: 10.1371/journal.pone.0013405 20976133 PMC2956635

[13] Kim D J, Noh J H, Lee B W, Choi Y H, Chung J H, Min Y K, et al. The associations of total and differential white blood cell counts with obesity, hypertension, dyslipidemia and glucose intolerance in a Korean population. J Korean Med Sci, 2008. 23(2): p. 193–8. doi: 10.3346/jkms.2008.23.2.193 18436999 PMC2526447

[14] Borné Y, Smith J G, Nilsson P M, Melander O, Hedblad B, and Engström G. Total and Differential Leukocyte Counts in Relation to Incidence of Diabetes Mellitus: A Prospective Population-Based Cohort Study. PLoS One, 2016. 11(2): p. e0148963. doi: 10.1371/journal.pone.0148963 26891449 PMC4758613

[15] Nagareddy P R, Murphy A J, Stirzaker R A, Hu Y, Yu S, Miller R G, et al. Hyperglycemia promotes myelopoiesis and impairs the resolution of atherosclerosis. Cell Metab, 2013. 17(5): p. 695–708. doi: 10.1016/j.cmet.2013.04.001 23663738 PMC3992275

[16] Flynn M C, Kraakman M J, Tikellis C, Lee M K S, Hanssen N M J, Kammoun H L, et al. Transient Intermittent Hyperglycemia Accelerates Atherosclerosis by Promoting Myelopoiesis. Circ Res, 2020. 127(7): p. 877–892. doi: 10.1161/CIRCRESAHA.120.316653 32564710 PMC7486277

[17] Astle W J, Elding H, Jiang T, Allen D, Ruklisa D, Mann A L, et al. The Allelic Landscape of Human Blood Cell Trait Variation and Links to Common Complex Disease. Cell, 2016. 167(5): p. 1415–1429.e19. doi: 10.1016/j.cell.2016.10.042 27863252 PMC5300907

[18] Li J, Niu Q, Wu A, Zhang Y, Hong L, and Wang H. Causal relationship between circulating immune cells and the risk of type 2 diabetes: a Mendelian randomization study. Front Endocrinol (Lausanne), 2023. 14: p. 1210415. doi: 10.3389/fendo.2023.1210415 37305035 PMC10247959

[19] Morris A P, Voight B F, Teslovich T M, Ferreira T, Segrè A V, Steinthorsdottir V, et al. Large-scale association analysis provides insights into the genetic architecture and pathophysiology of type 2 diabetes. Nat Genet, 2012. 44(9): p. 981–90. doi: 10.1038/ng.2383 22885922 PMC3442244

[20] Scott R A, Lagou V, Welch R P, Wheeler E, Montasser M E, Luan J, et al. Large-scale association analyses identify new loci influencing glycemic traits and provide insight into the underlying biological pathways. Nat Genet, 2012. 44(9): p. 991–1005. doi: 10.1038/ng.2385 22885924 PMC3433394

[21] Soranzo N, Sanna S, Wheeler E, Gieger C, Radke D, Dupuis J, et al. Common variants at 10 genomic loci influence hemoglobin A₁(C) levels via glycemic and nonglycemic pathways. Diabetes, 2010. 59(12): p. 3229–39. doi: 10.2337/db10-0502 20858683 PMC2992787

[22] Dupuis J, Langenberg C, Prokopenko I, Saxena R, Soranzo N, Jackson A U, et al. New genetic loci implicated in fasting glucose homeostasis and their impact on type 2 diabetes risk. Nat Genet, 2010. 42(2): p. 105–16. doi: 10.1038/ng.520 20081858 PMC3018764

[23] Lorenzo C, Hanley A J, and Haffner S M. Differential white cell count and incident type 2 diabetes: the Insulin Resistance Atherosclerosis Study. Diabetologia, 2014. 57(1): p. 83–92. doi: 10.1007/s00125-013-3080-0 24141640 PMC3969879

[24] Cai Z, Huang Y, and He B. New Insights into Adipose Tissue Macrophages in Obesity and Insulin Resistance. Cells, 2022. 11(9). doi: 10.3390/cells11091424 35563728 PMC9104938

[25] Kojta I, Chacińska M, and Błachnio-Zabielska A. Obesity, Bioactive Lipids, and Adipose Tissue Inflammation in Insulin Resistance. Nutrients, 2020. 12(5). doi: 10.3390/nu12051305 32375231 PMC7284998

[26] Calle M C and Fernandez M L. Inflammation and type 2 diabetes. Diabetes Metab, 2012. 38(3): p. 183–91. doi: 10.1016/j.diabet.2011.11.006 22252015

[27] Rehman K, Akash M S H, Liaqat A, Kamal S, Qadir M I, and Rasul A. Role of Interleukin-6 in Development of Insulin Resistance and Type 2 Diabetes Mellitus. Crit Rev Eukaryot Gene Expr, 2017. 27(3): p. 229–236. doi: 10.1615/CritRevEukaryotGeneExpr.2017019712 29199608

[28] Akbari M and Hassan-Zadeh V. IL-6 signalling pathways and the development of type 2 diabetes. Inflammopharmacology, 2018. 26(3): p. 685–698. doi: 10.1007/s10787-018-0458-0 29508109

[29] Akash M S H, Rehman K, and Liaqat A. Tumor Necrosis Factor-Alpha: Role in Development of Insulin Resistance and Pathogenesis of Type 2 Diabetes Mellitus. J Cell Biochem, 2018. 119(1): p. 105–110. doi: 10.1002/jcb.26174 28569437

