# Supplementary material for: White blood cells and type 2 diabetes: A Mendelian randomization study
Source: PLoS One. 2024 Mar 1;19(3):e0296701. doi: 10.1371/journal.pone.0296701 (PMC10906821; doi:10.1371/journal.pone.0296701)
Supplement: S1 Fig — (PDF) [file pone.0296701.s001.pdf]

S1 Figure

Figure S1-1 Forest plot of univariable MR analysis for the causal association between white blood cells and type 2 diabetes (T2D).

A causal estimation of neutrophil on T2DM

B causal estimation of lymphocyte on T2DM

C causal estimation of monocyte on T2DM

D causal estimation of eosinophil on T2DM

E causal estimation of basophil on T2DM

A

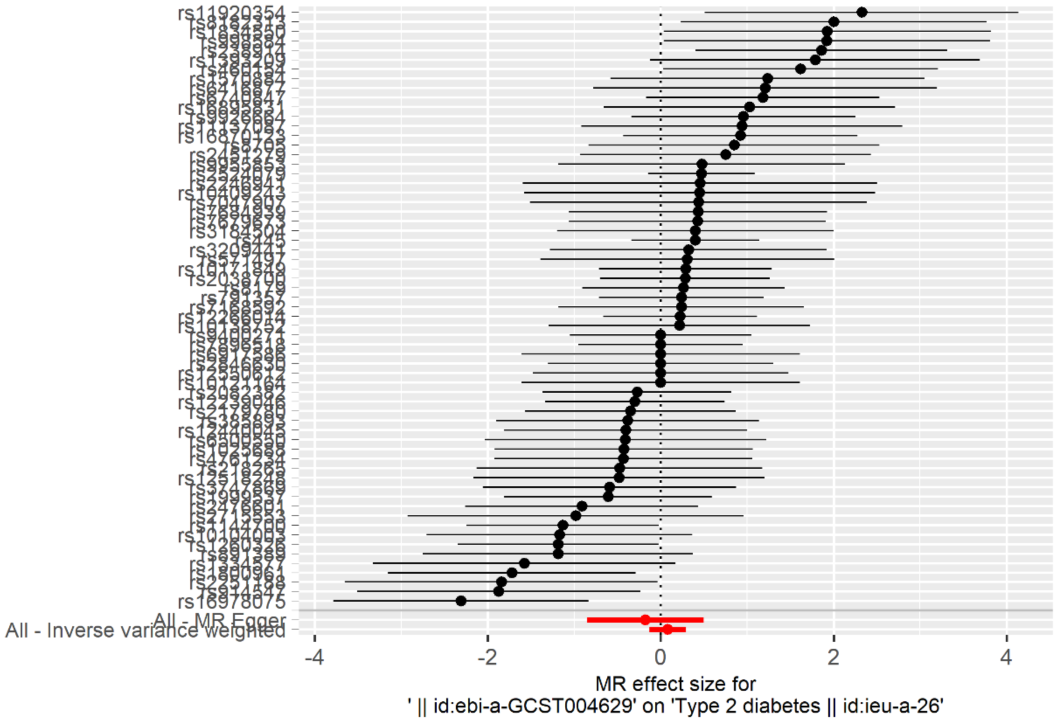

B

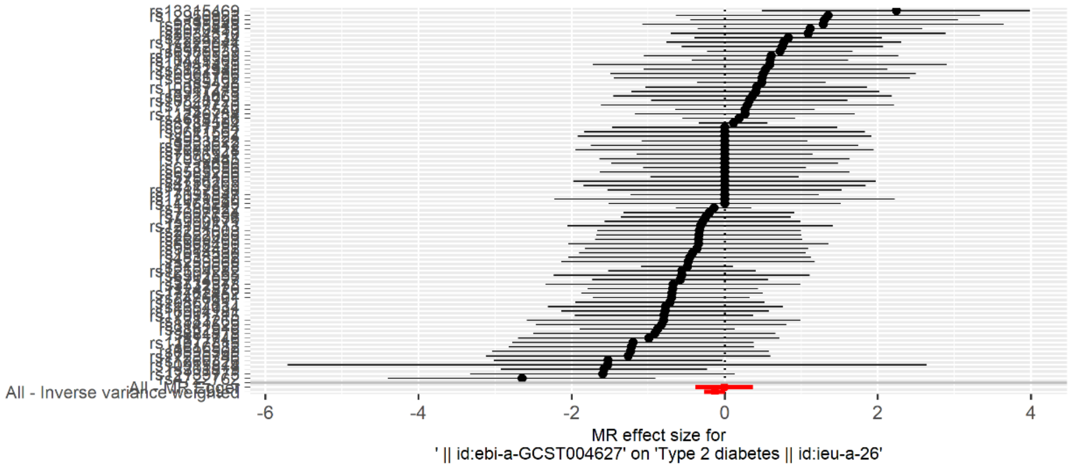

C

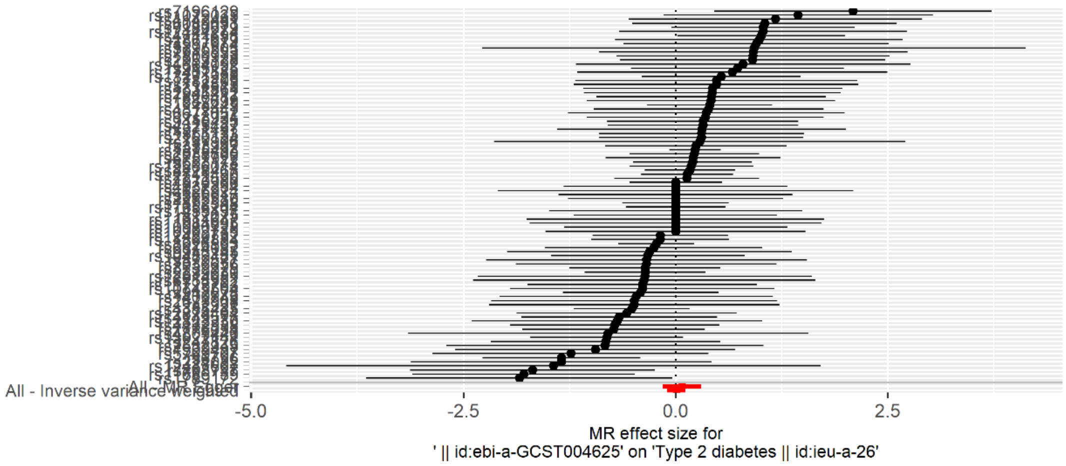

D

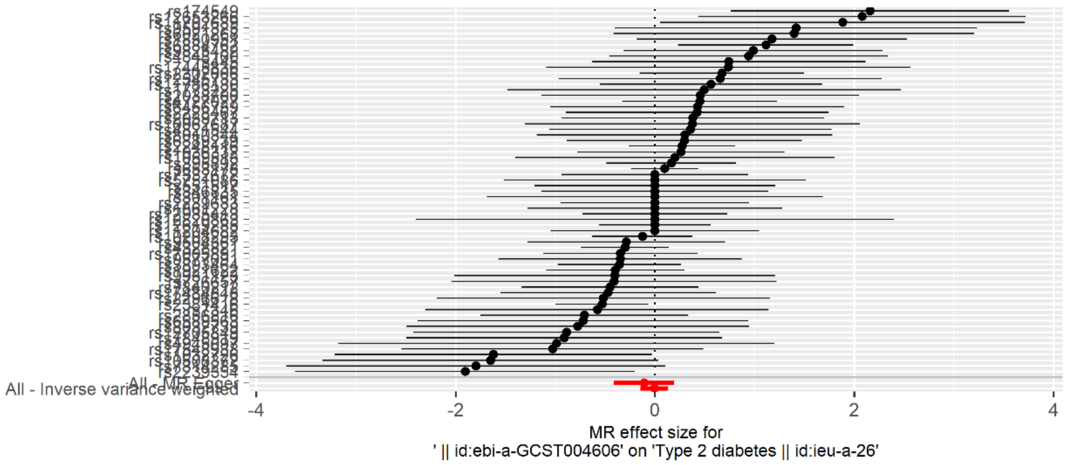

E

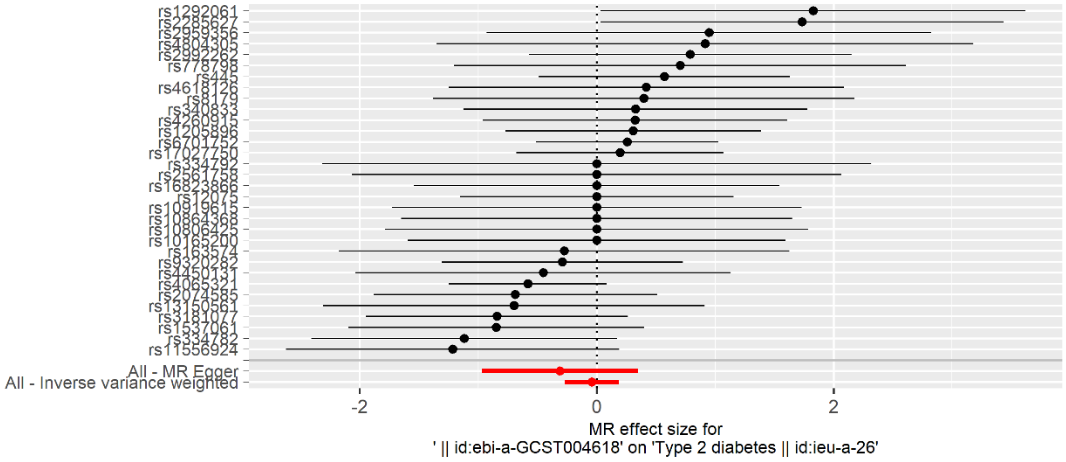

Figure S1-2 Funnel plot of univariable MR analysis for the causal association between white blood cells and type 2 diabetes (T2DM).

A causal estimation of neutrophil on T2DM

B causal estimation of lymphocyte on T2DM

C causal estimation of monocyte on T2DM

D causal estimation of eosinophil on T2DM

E causal estimation of basophil on T2DM

A

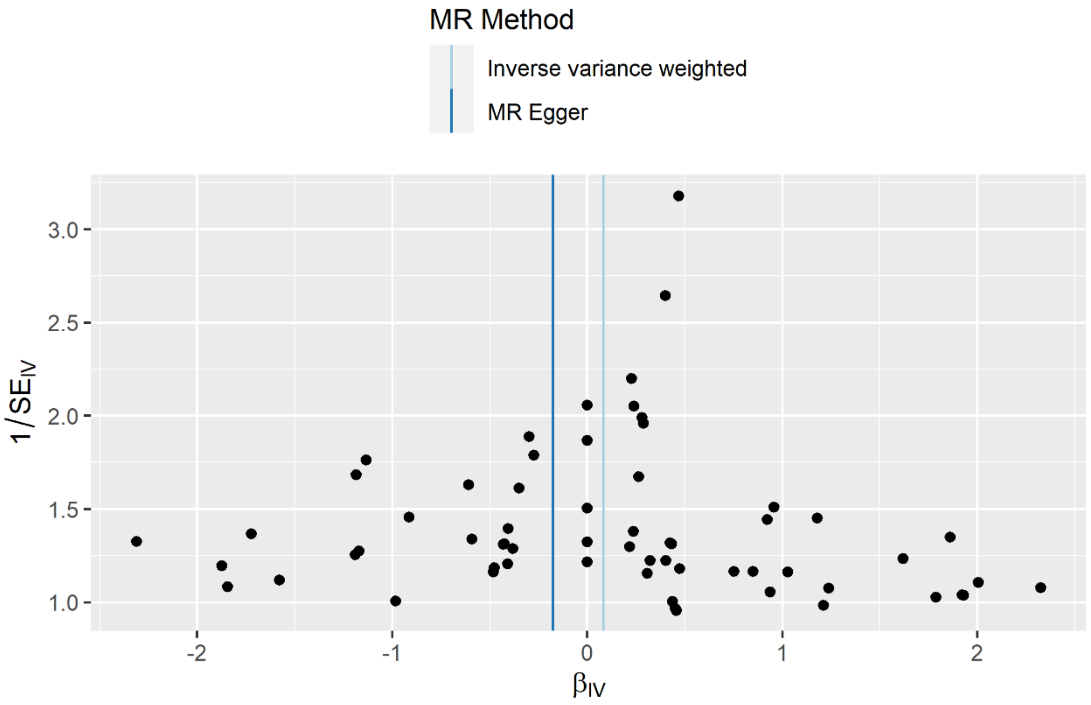

B

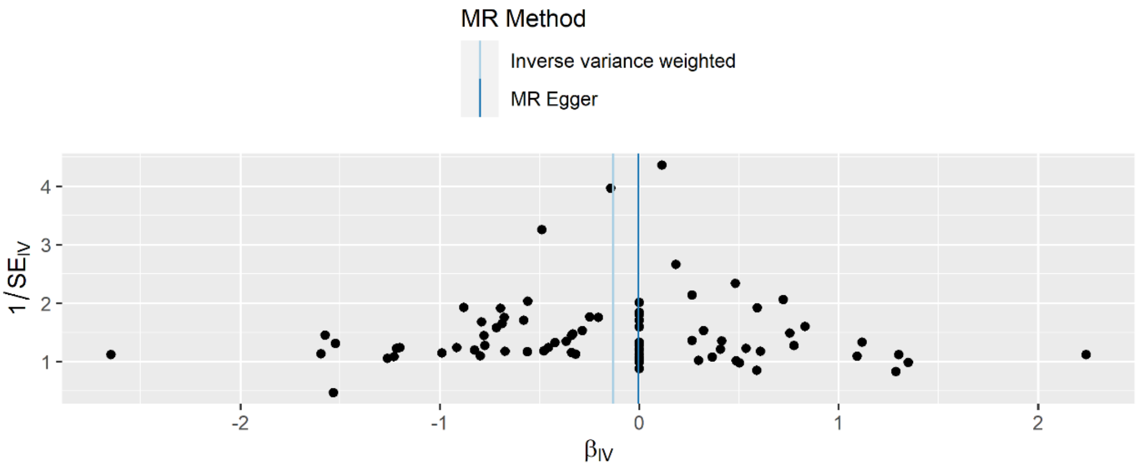

C

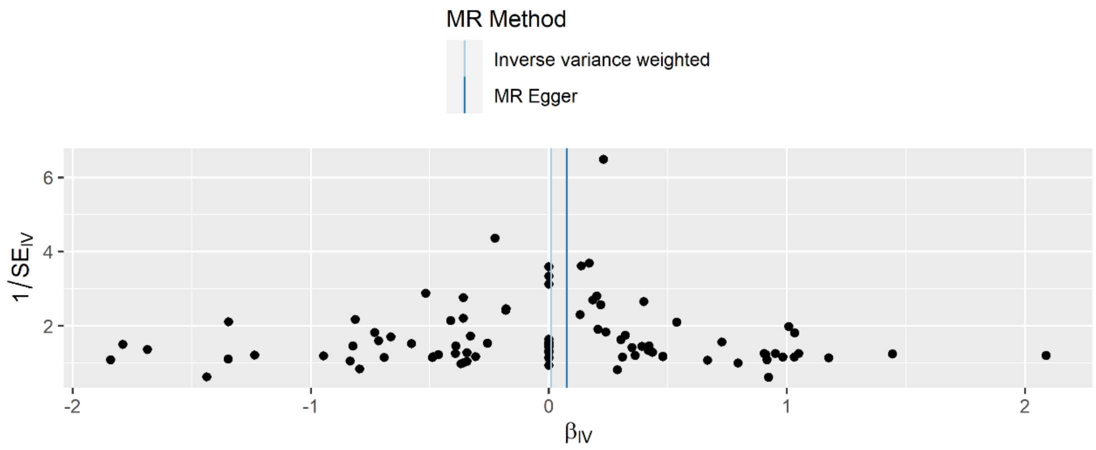

D

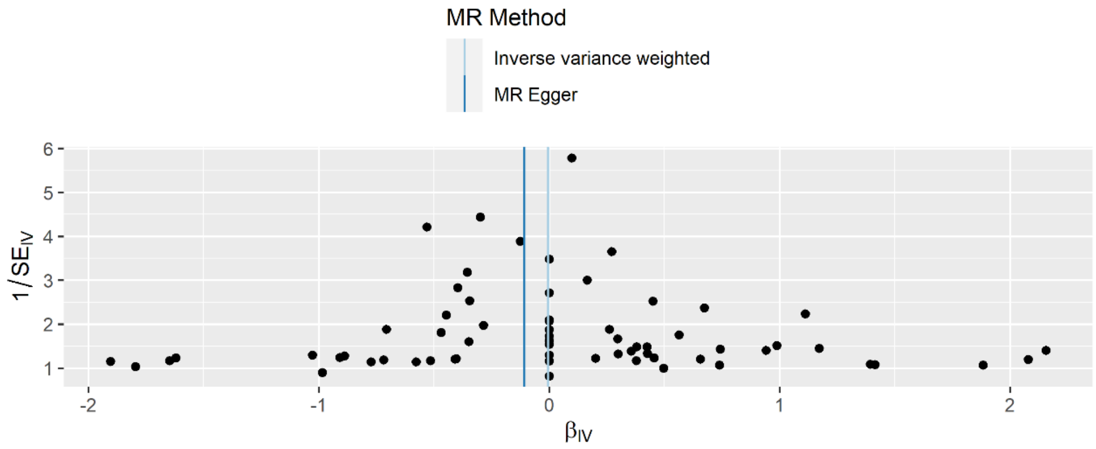

E

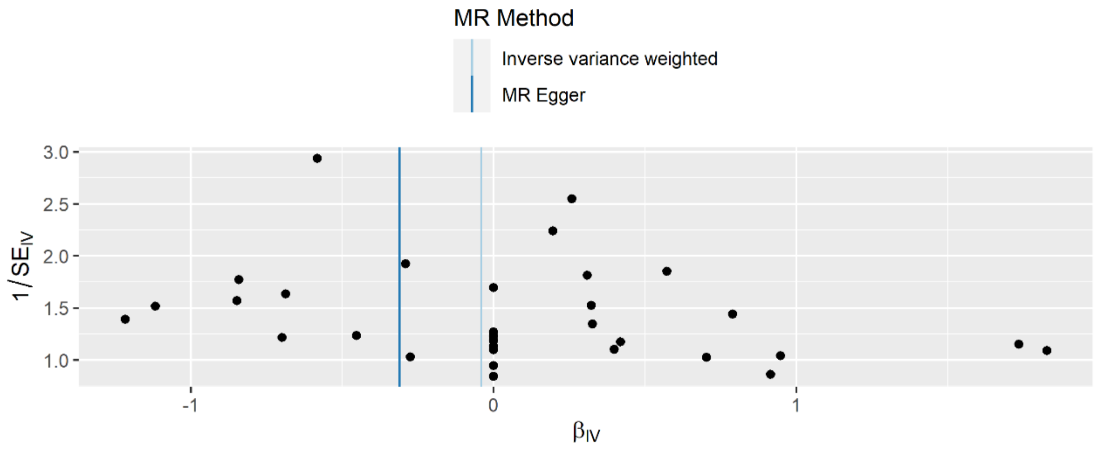

Figure S1-3 Scatter plot of univariable MR analysis for the causal association between white blood cells and type 2 diabetes (T2DM).

A causal estimation of neutrophil on T2DM

B causal estimation of lymphocyte on T2DM

C causal estimation of monocyte on T2DM

D causal estimation of eosinophil on T2DM

E causal estimation of basophil on T2DM

A

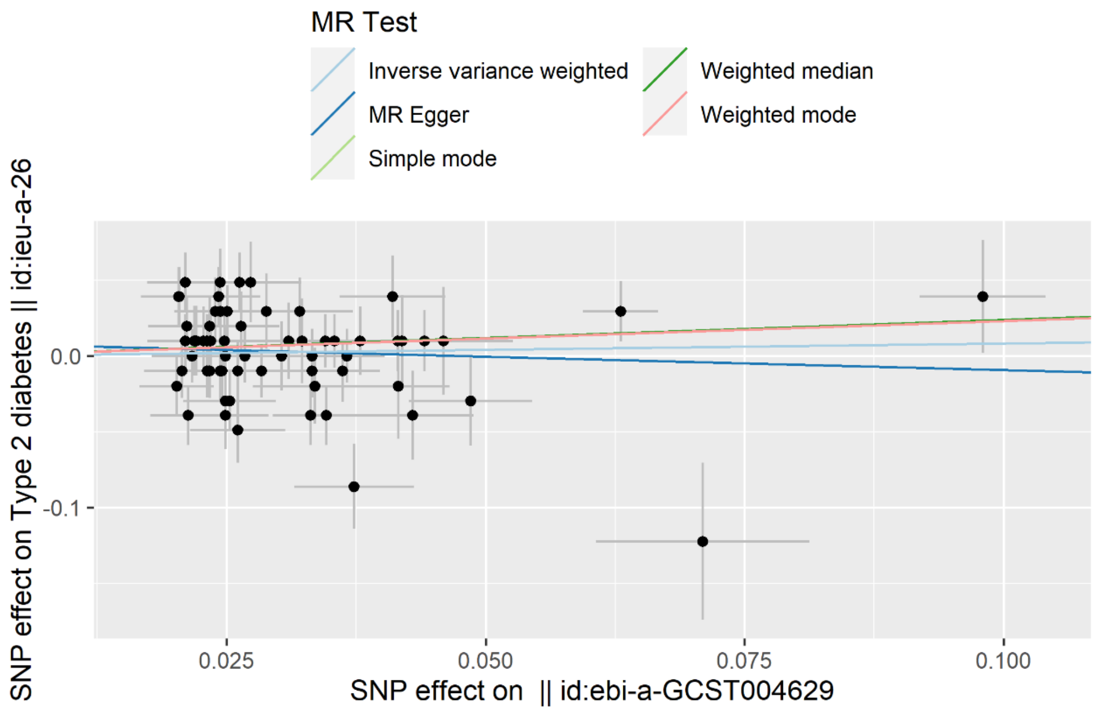

B

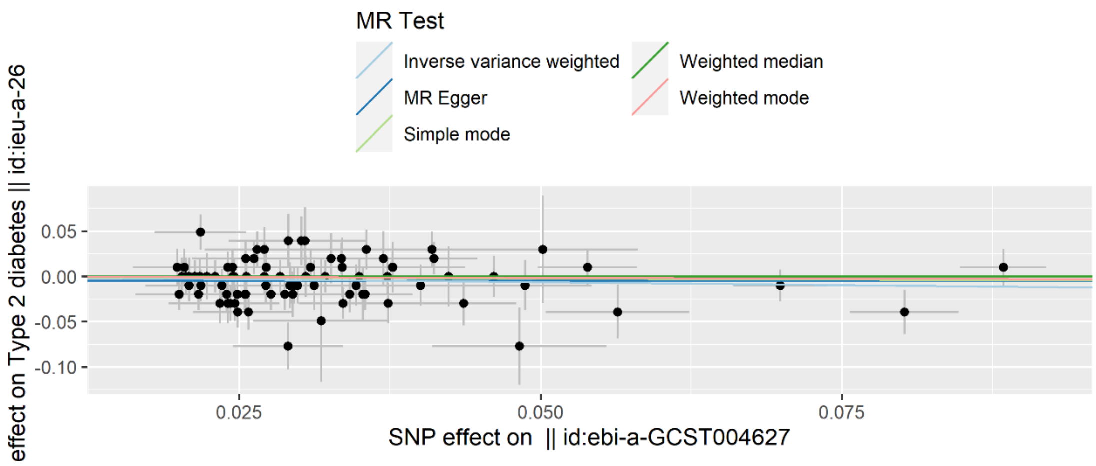

C

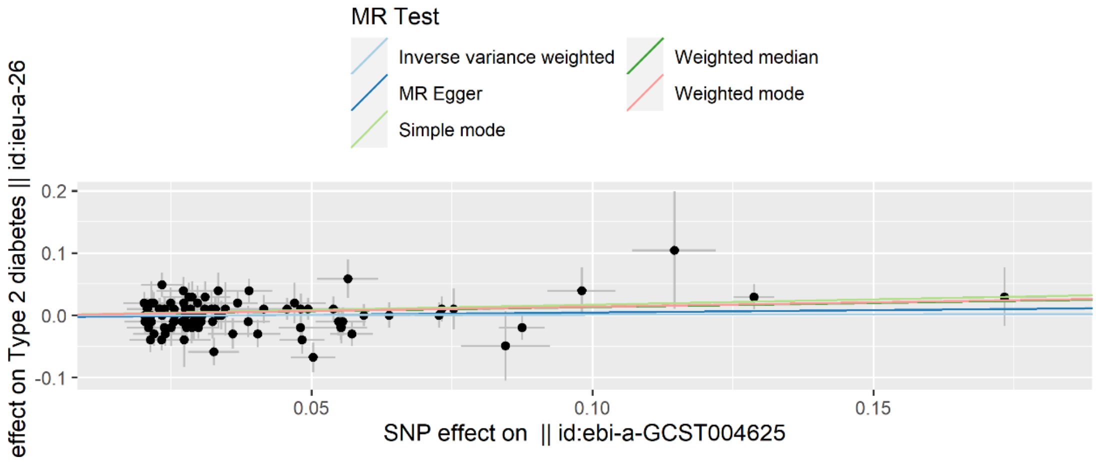

D

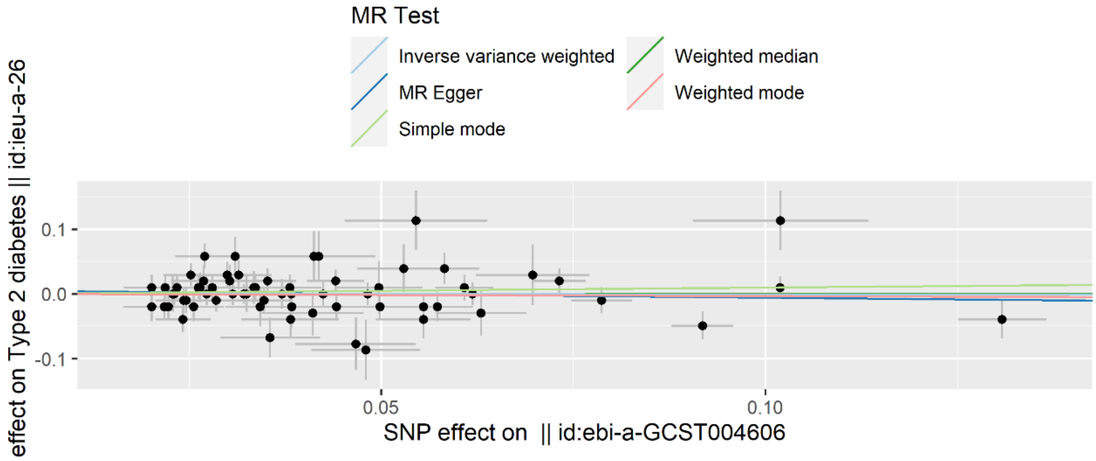

E

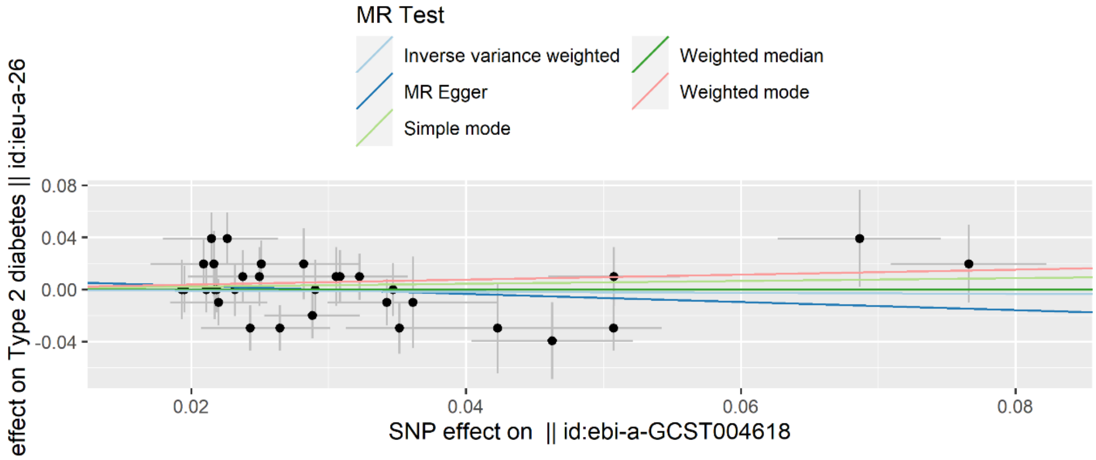

Figure S1-4 Leave-one-out analysis of univariable MR for the causal association between white blood cells and type 2 diabetes (T2DM).

A causal estimation of neutrophil on T2DM

B causal estimation of lymphocyte on T2DM

C causal estimation of monocyte on T2DM

D causal estimation of eosinophil on T2DM

E causal estimation of basophil on T2DM

A

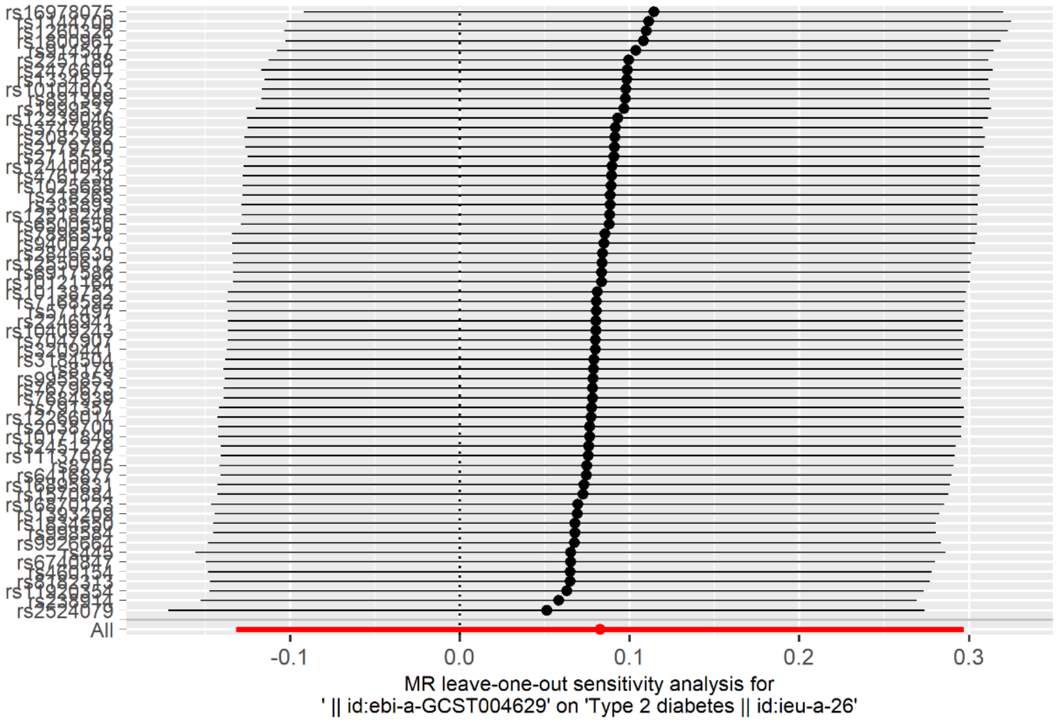

B

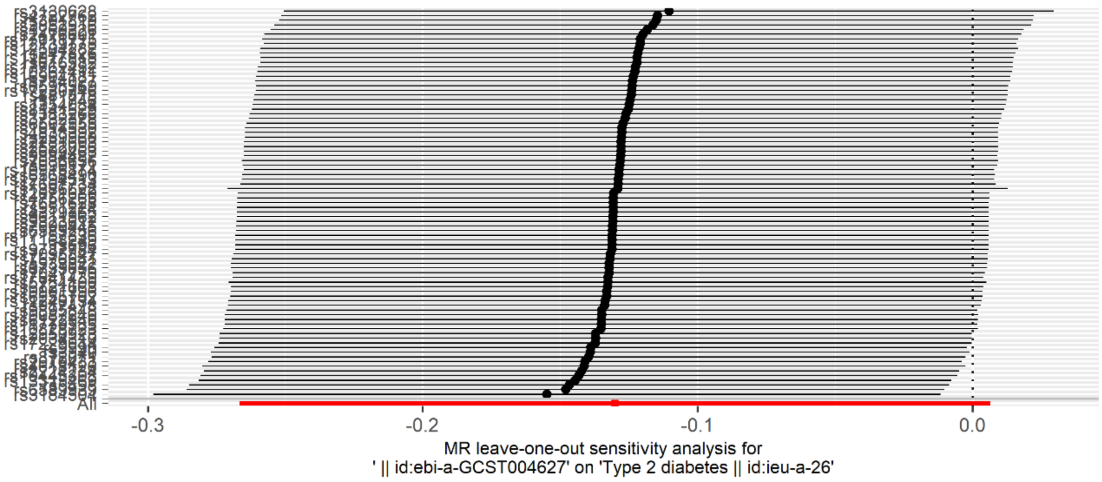

C

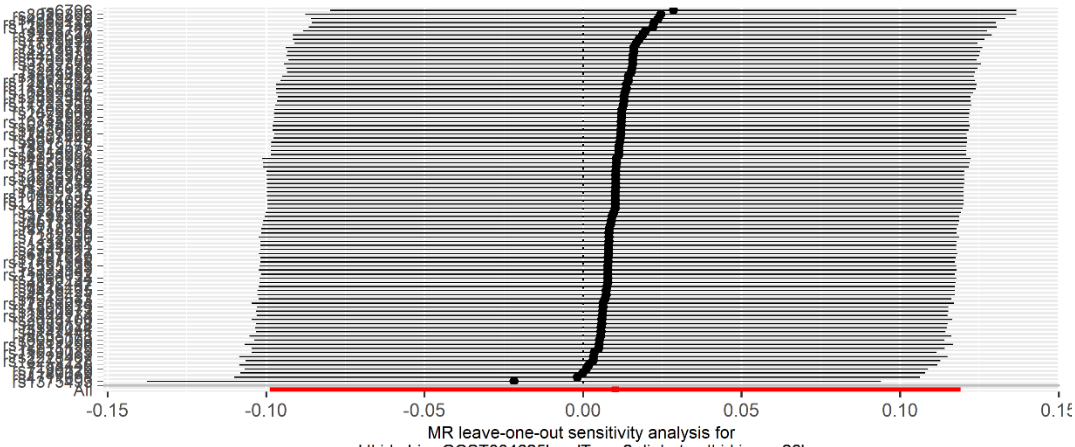
MR leave-one-out sensitivity analysis for
' || id:ebi-a-GCST004625' on 'Type 2 diabetes || id:ieu-a-26'

D

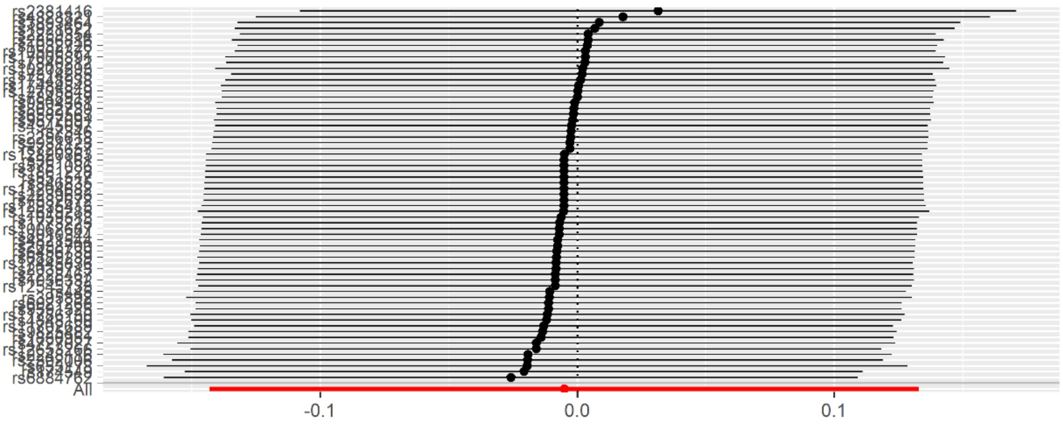
MR leave-one-out sensitivity analysis for
' || id:ebi-a-GCST004606' on 'Type 2 diabetes || id:ieu-a-26'

E

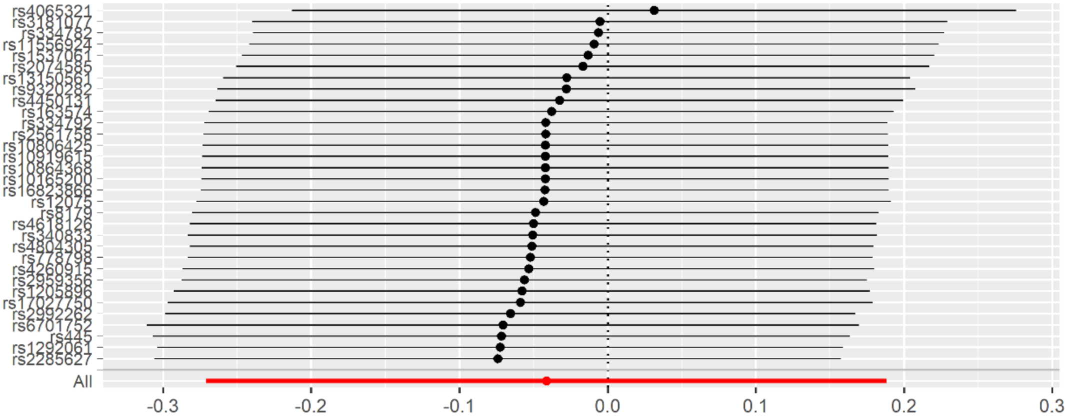
MR leave-one-out sensitivity analysis for
' || id:ebi-a-GCST004618' on 'Type 2 diabetes || id:ieu-a-26'
