# Supplementary material for: White blood cells and type 2 diabetes: A Mendelian randomization study
Source: PLoS One. 2024 Mar 1;19(3):e0296701. doi: 10.1371/journal.pone.0296701 (PMC10906821; doi:10.1371/journal.pone.0296701)
Supplement: S2 Table — (DOC) [file pone.0296701.s003.doc]

**S2 Table**

**Table S2-1 Results of the heterogeneity test and pleiotropy test of the causal effect of white blood cells on FG.**

| Exposure | Outcome | NSNP | F-statistics | heterogeneity | | pleiotropy | |
| --- | --- | --- | --- | --- | --- | --- | --- |
|  |  |  | Mean  (min-max) | methods | Cochran’s Q statistics (*P*) | MR-Egger intercept  (*P*) | MR-PRESSO  outlier |
| Neutrophil | Fasting glucose | 6 | 90.77 (32.16-301.76) | MR-Egger | 5.24 (0.263) | -0.001 (0.842) | 3 SNPs  (rs1260326  rs2476601  rs7896518) |
|  |  |  |  | IVW | 5.30 (0.380) |  |  |
| Lymphocyte | Fasting glucose | 7 | 64.67 (48.64-128.84) | MR-Egger | 8.37 (0.137) | -0.002 (0.783) | 3 SNPs  (rs1260326  rs2476601  rs3184504) |
|  |  |  |  | IVW | 8.51 (0.203) |  |  |
| Monocyte | Fasting glucose | 6 | 97.29 (31.04-191.94) | MR-Egger | 7.49 (0.112) | -0.001 (0.884) | No SNP |
|  |  |  |  | IVW | 7.54 (0.184) |  |  |
| Eosinophil | Fasting glucose | 7 | 182.82 (29.90-821.43) | MR-Egger | 6.83 (0.233) | -3.78e-05  (0.987) | 1 SNP  (rs174549) |
|  |  |  |  | IVW | 6.83 (0.337) |  |  |
| Basophil | Fasting glucose | 2 | 39.68 (33.37-46.00) | MR-Egger | NA | NA |  |
|  |  |  |  | IVW | 3.88 (0.049) |  |  |

**Table S2-2 Results of the heterogeneity test and pleiotropy test of the causal effect of white blood cells on HbA1c.**

| Exposure | Outcome | NSNP | F-statistics | heterogeneity | | pleiotropy | |
| --- | --- | --- | --- | --- | --- | --- | --- |
|  |  |  | Mean  (min-max) | methods | Cochran’s Q statistics (*P*) | MR-Egger intercept  (*P*) | MR-PRESSO  outlier |
| Neutrophil | HbA1c | 61 | 61.00 (30.78-306.26) | MR-Egger | 79.60 (0.038) | -0.003 (0.199) | 2 SNPs  (rs3184504  rs9400271) |
|  |  |  |  | IVW | 81.88 (0.032) |  |  |
| Lymphocyte | HbA1c | 80 | 60.32 (29.87-323.88) | MR-Egger | 104.59 (0.024) | 0.001 (0.532) | 4 SNPs  (rs2157691  rs3184504  rs3818717  rs7040409) |
|  |  |  |  | IVW | 105.11 (0.026) |  |  |
| Monocyte | HbA1c | 108 | 100.16 (30.59-1280.97) | MR-Egger | 128.96 (0.064) | 1.109e-5  (0.995) | No SNP |
|  |  |  |  | IVW | 128.96 (0.073) |  |  |
| Eosinophil | HbA1c | 65 | 98.00 (29.90-520.00) | MR-Egger | 77.65 (0.101) | -0.001  (0.572) | 1 SNP  (rs9320282) |
|  |  |  |  | IVW | 78.05 (0.111) |  |  |
| Basophil | HbA1c | 31 | 58.70 (29.81-209.21) | MR-Egger | 52.33 (0.005) | 0.001  (0.793) | 1 SNP  (rs9320282) |
|  |  |  |  | IVW | 52.45 (0.007) |  |  |

**Table S2-3 Results of the heterogeneity test and pleiotropy test of the causal effect of white blood cells on HOMA-IR.**

| Exposure | Outcome | NSNP | F-statistics | heterogeneity | | pleiotropy | |
| --- | --- | --- | --- | --- | --- | --- | --- |
|  |  |  | Mean  (min-max) | methods | Cochran’s Q statistics (*P*) | MR-Egger intercept  (*P*) | MR-PRESSO  outlier |
| Neutrophil | HOMA-IR | 59 | 58.91 (30.78-306.26) | MR-Egger | 84.00 (0.012) | 3.16*10^-4^  (0.900) | 1 SNP  (rs1260326) |
|  |  |  |  | IVW | 84.02 (0.014) |  |  |
| Lymphocyte | HOMA-IR | 81 | 67.07 (29.87-606.36) | MR-Egger | 85.41 (0.291) | -0.001  (0.517) | 1 SNP  (rs1260326) |
|  |  |  |  | IVW | 85.87 (0.307) |  |  |
| Monocyte | HOMA-IR | 85 | 105.39 (30.75-1280.97) | MR-Egger | 113.86 (0.014) | -0.002 (0.155) | No SNP |
|  |  |  |  | IVW | 116.69 (0.011) |  |  |
| Eosinophil | HOMA-IR | 64 | 110.77 (29.90-821.43) | MR-Egger | 88.03 (0.017) | -0.001  (0.599) | No SNP |
|  |  |  |  | IVW | 88.43 (0.019) |  |  |
| Basophil | HOMA-IR | 31 | 60.66 (29.81-209.21) | MR-Egger | 22.12 (0.815) | -0.001  (0.701) | No SNP |
|  |  |  |  | IVW | 22.27 (0.844) |  |  |
